# Photothermal Prussian blue nanoparticles generate potent multi‐targeted tumor‐specific T cells as an adoptive cell therapy

**DOI:** 10.1002/btm2.10639

**Published:** 2023-12-22

**Authors:** Elizabeth E. Sweeney, Palak Sekhri, Nethaji Muniraj, Jie Chen, Sally Feng, Joshua Terao, Samantha J. Chin, Danielle E. Schmidt, Catherine M. Bollard, Conrad Russell Y. Cruz, Rohan Fernandes

**Affiliations:** ^1^ Department of Biochemistry & Molecular Medicine, School of Medicine and Health Sciences George Washington University Washington District of Columbia USA; ^2^ Center for Cancer and Immunology Research Children's National Hospital Washington District of Columbia USA; ^3^ The Integrated Biomedical Sciences Program, School of Medicine and Health Sciences George Washington University Washington District of Columbia USA; ^4^ George Washington Cancer Center, School of Medicine and Health Sciences George Washington University Washington District of Columbia USA; ^5^ Department of Medicine, School of Medicine and Health Sciences George Washington University Washington District of Columbia USA

**Keywords:** adoptive T cell therapy, cancer, hematological malignancies, photothermal therapy, Prussian blue nanoparticles, solid tumors, tumor‐specific T cells

## Abstract

Prussian blue nanoparticle‐based photothermal therapy (PBNP‐PTT) is an effective tumor treatment capable of eliciting an antitumor immune response. Motivated by the ability of PBNP‐PTT to potentiate endogenous immune responses, we recently demonstrated that PBNP‐PTT could be used ex vivo to generate tumor‐specific T cells against glioblastoma (GBM) cell lines as an adoptive T cell therapy (ATCT). In this study, we further developed this promising T cell development platform. First, we assessed the phenotype and function of T cells generated using PBNP‐PTT. We observed that PBNP‐PTT facilitated CD8+ T cell expansion from healthy donor PBMCs that secreted IFNγ and TNFα and upregulated CD107a in response to engagement with target U87 cells, suggesting specific antitumor T cell activation and degranulation. Further, CD8+ effector and effector memory T cell populations significantly expanded after co‐culture with U87 cells, consistent with tumor‐specific effector responses. In orthotopically implanted U87 GBM tumors in vivo, PBNP‐PTT‐derived T cells effectively reduced U87 tumor growth and generated long‐term survival in >80% of tumor‐bearing mice by Day 100, compared to 0% of mice treated with PBS, non‐specific T cells, or T cells expanded from lysed U87 cells, demonstrating an enhanced antitumor efficacy of this ATCT platform. Finally, we tested the generalizability of our approach by generating T cells targeting medulloblastoma (D556), breast cancer (MDA‐MB‐231), neuroblastoma (SH‐SY5Y), and acute monocytic leukemia (THP‐1) cell lines. The resulting T cells secreted IFNγ and exerted increased tumor‐specific cytolytic function relative to controls, demonstrating the versatility of PBNP‐PTT in generating tumor‐specific T cells for ATCT.

**Translational Impact Statement**

This study is translationally relevant because it expands on a prior proof‐of‐concept study demonstrating the use of PBNP‐PTT to generate tumor‐specific T cells as an adoptive T cell therapy (ATCT). Based on the promising findings in vivo and in multiple tumor cell types, we envision advancing the technology to generate personalized ATCT for multiple cancer indications, complementing the current milieu of T cell products available to patients, including chimeric antigen receptor (CAR) T cells and tumor‐associated antigen‐specific (TAA) T cells.

## 1 INTRODUCTION

Prussian blue nanoparticle (PBNP)‐based photothermal therapy (PBNP‐PTT) represents an effective light activated, nanoparticle‐mediated tumor treatment.^1^, ^2^, ^3^ PBNPs are face‐centered cubic lattice structures^4^ that have been extensively studied by others^1^, ^5^, ^6^, ^7^, ^8^, ^9^, ^10^ and us^2^, ^3^, ^11^, ^12^, ^13^, ^14^, ^15^, ^16^, ^17^, ^18^, ^19^, ^20^, ^21^ for their use as agents of photothermal therapy for cancer. PBNPs strongly absorb light in the near infrared (NIR) range,^22^ and generate heat when illuminated by wavelengths matching this absorption spectrum.^2^, ^20^ PBNP‐PTT generates a potent antitumor immune response in tumor‐bearing animals, especially when combined with immune adjuvants and immunotherapy.^15^, ^16^, ^17^, ^19^, ^23^ Our group has demonstrated that PBNP‐PTT decreases tumor burden and increases survival in mouse models of several solid tumors.^2^, ^3^, ^11^, ^12^, ^16^, ^19^ These therapeutic benefits were driven by T cell infiltration into tumors and specific antitumor T cell activation and cytotoxicity.^2^, ^15^, ^16^, ^17^, ^19^ We discovered that at certain thermal doses, PBNP‐PTT elicits immunogenic cell death (ICD),^20^ characterized by the recruitment and engagement of antigen‐presenting cells (e.g., dendritic cells (DCs)) for subsequent T cell activation.^24^, ^25^, ^26^

**SCHEME 1:**
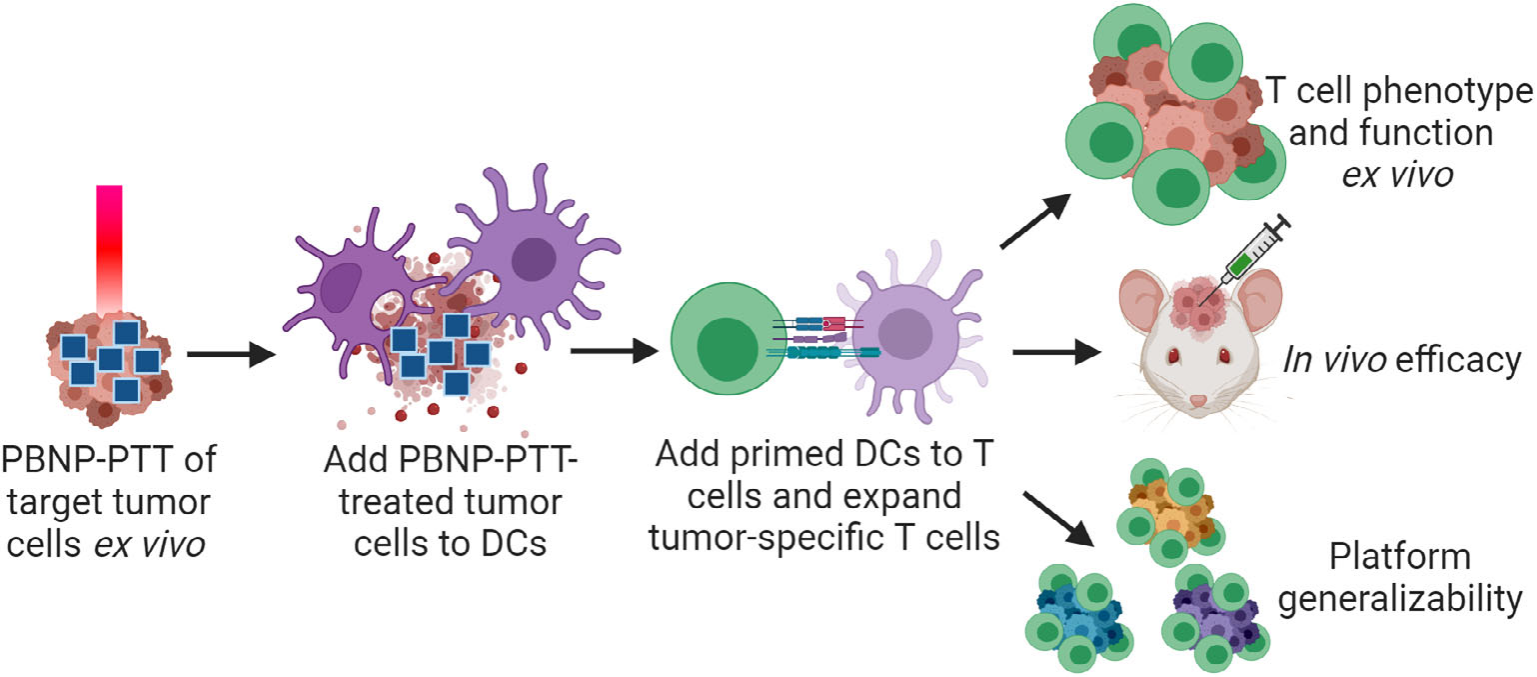
PBNP‐PTT as a platform for ex vivo tumor‐specific T cell expansion. The PBNP‐PTT‐derived T cell expansion scheme is illustrated, where PBNP‐PTT is administered to target tumor cells ex vivo (0.15 mg/mL PBNPs and 1.5 W 808 nm laser treatment for 10 min). Next, treated tumor cells are administered to dendritic cells (DCs) matched to the tumor cells at HLA‐A. Primed DCs are next added to autologous T cells to expand a tumor‐specific T cell population. Here, we characterize the function of PBNP‐PTT‐derived T cells by (1) co‐culturing the T cells with the target tumor cells and measuring T cell phenotype and cytokine secretion ex vivo, (2) treating established tumors in vivo, in comparison to other T cell products and controls, and (3) evaluating the applicability of the PBNP‐PTT‐mediated T cell expansion platform to other tumor types (i.e., medulloblastoma, breast cancer, neuroblastoma, and acute monocytic leukemia) ex vivo.

Based on the ability of PBNP‐PTT to induce immunogenicity and potentiate endogenous immune responses in vivo, we have developed a PBNP‐PTT‐based expansion scheme to generate tumor‐specific T cells ex vivo as an adoptive T cell therapy (ATCT) product (Scheme 1). In this ex vivo expansion scheme, PBNP‐PTT is used to heat, kill, and induce immunogenicity of target tumor cells,^18^, ^20^ such that DCs co‐cultured with PBNP‐PTT‐treated tumor cells exhibit tumor antigen uptake and presentation, and in response, facilitate the expansion of tumor‐specific T cells. Although we are only using PBNPs ex vivo in the preparation of the ATCT, it is important to note that PBNPs are biodegradable in physiologic conditions,^16^ and are approved by the FDA for human oral use,^27^ suggesting a suitable safety profile for use in this application. In a recent proof‐of‐concept study, we demonstrated that PBNP‐PTT can be used to generate glioblastoma (GBM) cell line (U87)‐specific T cells.^21^ ATCT, including chimeric antigen receptor (CAR) T cell therapy, is a promising strategy for treating cancers resistant to standard therapies.^28^, ^29^, ^30^, ^31^, ^32^, ^33^, ^34^, ^35^, ^36^ Hematological malignancies have been treated successfully with ATCT,^28^, ^29^, ^37^, ^38^ while solid tumors, including brain tumors, have been more difficult to treat with these approaches.^39^, ^40^ One feature that may contribute to treatment failure is that CAR T cells and tumor‐associated antigen (TAA)‐specific T cells are often designed against one or a few defined antigen targets.^30^, ^41^ Targeting a single or few antigens in a heterogeneous solid tumor inevitably leads to immune escape, an increasing source of CAR T cell therapy failure.^42^ In the context of GBM, a focus of this study, the intrinsic heterogeneity is a critical limiting factor and the basis of treatment failure using ATCT approachs.^43^, ^44^, ^45^, ^46^, ^47^, ^48^

Therefore, we hypothesize that PBNP‐PTT is an effective method to generate an expanded source of multiple unique antigens present in GBM. Consequently, T cells expanded using PBNP‐PTT may effectively target a variety of tumor antigens, thereby addressing the cellular heterogeneity responsible for therapeutic resistance of GBM and other solid tumors.^21^, ^49^ Unlike T cells generated from cell lysis approaches, which rely on directly lysing the tumor cells to expand T cells to multiple tumor antigens,^21^, ^50^, ^51^ ICD elicited by PBNP‐PTT is expected to facilitate enhanced antigen uptake and presentation, thereby generating an improved tumor‐specific T cell population compared to direct lysis of tumor cells. In our previous paper,^21^ we generated T cells targeted to two GBM cell lines, U87 and SNB19, using healthy donor peripheral blood mononuclear cells (PBMCs) matched to the tumor cell lines at HLA‐A*02. Importantly, the T cells developed via PBNP‐PTT specifically and significantly secreted IFNγ in response to the target GBM cells in a dose‐dependent manner, and specifically killed target GBM cells after a 4 h co‐culture, while sparing normal human astrocytes (NHA).^21^

Building on our earlier findings, in this study, we characterize GBM‐specific T cells developed via PBNP‐PTT (Scheme 1). We describe the relevant immune cell subsets present in the U87‐specific T cell product. Since the healthy donor PBMCs used to expand the T cells were matched to the target tumor cells at HLA‐A, we focus on the phenotype and function of the CD8+ T cell population upon interaction with the target cells. To evaluate the advantage of PBNP‐PTT, we compare T cells expanded via PBNP‐PTT with T cells expanded from the same donor using U87 cell lysates. Next, we test the efficacy of PBNP‐PTT‐derived T cells in an orthotopic xenograft GBM model in vivo in comparison to non‐specific or lysate‐derived T cells. Finally, we test the applicability of the PBNP‐PTT‐based platform to generate tumor‐specific T cells targeted to other cellular models of solid and hematological malignancies. These studies will provide the rationale for future development of the PBNP‐PTT‐based T cell platform to translate novel ATCTs for patients with cancer resistant to standard therapies, complementing alternative ATCT approaches currently under clinical evaluation.^52^, ^53^

## 2 RESULTS

### 2.1 PBNP‐PTT generates U87‐specific T cells, evidenced by the CD8+ T cell subset which activate and degranulate in response to engagement with target U87 cells

We used PBNP‐PTT to generate U87‐specific T cells (*n* = 2 donors) by administering PBNP‐PTT to the U87 tumor cells, adding them to DCs, and subsequently using these primed DCs to expand tumor‐specific T cells (Scheme 1; Methods for details). We analyzed the phenotype and function of the resulting PBNP‐PTT‐derived T cells and compared them to T cells generated by directly lysing tumor cells (Lysate‐derived T cells) and the unexpanded CD14− population of the PBMCs (Native) from which the T cells were expanded.

**FIGURE 1:**
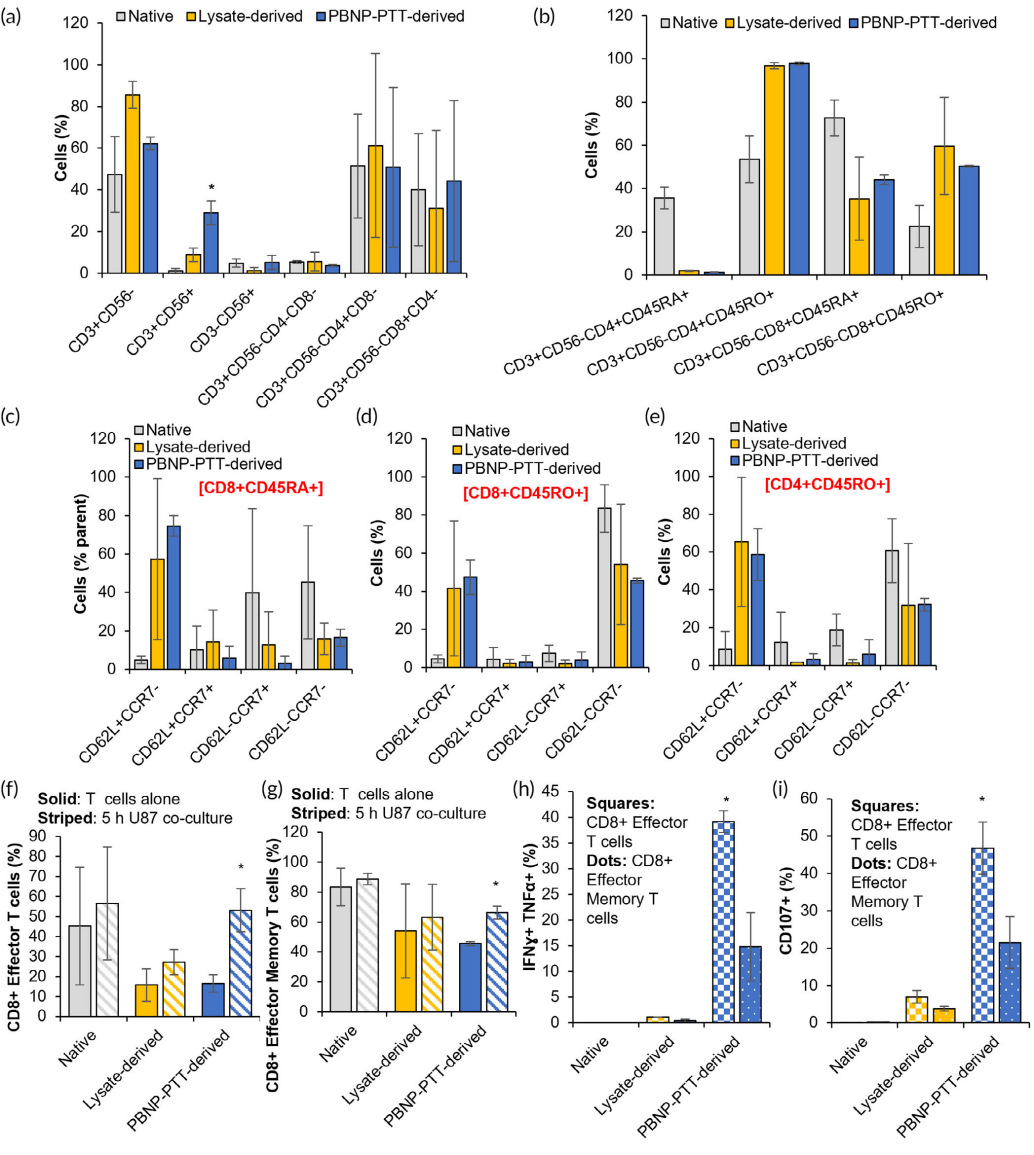
PBNP‐PTT generates effective U87‐specific T cells evidenced by the CD8+ T cell subset, which activate and degranulate in response to engagement with target U87 cells. CD14− cells were isolated from healthy donor PBMCs (*n* = 2 donors) and immediately analyzed (Native; gray) or allowed to expand using U87 cell lysate‐mediated (Lysate‐derived; yellow) or PBNP‐PTT‐mediated (PBNP‐PTT‐derived; blue) manufacture techniques. (a–e) Cells were analyzed for phenotype by flow cytometry. Values represent means (*n* = 2 donors/group) ± standard deviation. (f–i) Native CD14− cells (Native; gray) or T cells generated via U87 cell lysate‐mediated (Lysate‐derived; yellow) or PBNP‐PTT‐mediated (PBNP‐PTT‐derived; blue) manufacture techniques were co‐cultured with U87 cells at an E:T ratio of 1:1. After 5 h, T cells were analyzed for (f, g) phenotype, (h) intracellular expression of IFNγ and TNFα, and (i) expression of CD107a. Values represent means (*n* = 2 donors/group) ± standard deviation. *p < 0.05. Effector T cells are identified as CD45RA+CD62L−CCR7−. Effector Memory T cells are identified as CD45RO+CD62L−CCR7−.

Native, Lysate‐derived, and PBNP‐PTT‐derived T cell products comprised an average of 47.4 ± 18.1, 85.5 ± 6.4, and 62.2 ± 3.1% CD3+CD56− cells, which we identify as T cells (Figure 1a). Notably, PBNP‐PTT‐derived cells comprised a significantly higher proportion of CD3 + CD56+ cells (29.0 ± 5.6%) versus Native (1.1 ± 1.2%) and Lysate‐derived (8.9 ± 3.2%) cell products. Of the T cells (CD3+CD56−), all products had statistically identical populations of CD3−CD56+ (identified as NK cells), CD3+CD56−CD4−CD8− cells, and CD4+/CD8+ T cells.

Next, T cells were evaluated for CD45RA and CD45RO expression, as a measure of antigen experience.^54^ Of the CD4+ T cells, Native, Lysate‐derived, and PBNP‐PTT‐derived cell products comprised an average of 35.7 ± 5.0, 2.0 ± 0.3, and 1.3 ± 0.1% CD45RA+ cells, and 53.6 ± 10.9, 96.9 ± 1.5, and 98.0 ± 0.6% CD45RO+ cells, respectively (Figure 1b), suggesting the expansion of memory CD4+ T cell populations in the Lysate‐derived and PBNP‐PTT‐derived products. Of the CD8+ T cells, Native, Lysate‐derived, and PBNP‐PTT‐derived T cell products comprised an average of 72.7 ± 8.3, 35.3 ± 19.2, and 44.1 ± 2.2% CD45RA+ cells, and 22.5 ± 9.8, 59.7 ± 22.5, and 50.3 ± 0.4% CD45RO+ cells, respectively (Figure 1b), suggesting the expansion of memory CD8+ T cell populations in the Lysate‐derived and PBNP‐PTT‐derived products.^55^

Of the CD8+ antigen‐naïve (CD45RA+) T cells, all manufacturing methods generated statistically equivalent populations of Effector T cells (as identified as CD62L−CCR7−) (Figure 1c). Of CD8+ and CD4+ antigen‐experienced (CD45RO+) T cells, all manufacturing methods generated statistically equivalent populations of Effector Memory T cells (as identified as CD62L−CCR7−) and Central Memory T cells (as identified as CD62L+CCR7+) (Figure 1d,e).

To determine whether the cells resulting from U87 lysate‐ or PBNP‐PTT‐mediated expansion respond to or engage with the target U87 cells, indicative of target tumor specificity, T cells were co‐cultured with U87 cells for 5 h, and then the cells were characterized for phenotype. After encountering the U87 cells, PBNP‐PTT‐derived T cells significantly increased the proportion of CD8+ effector T cells (CD3+CD56−CD8+CD45RA+CD62L−CCR7−) from 16.5 ± 4.5% to 53.2 ± 10.8%, and effector memory (CD3+CD56−CD8+CD45RO+CD62L−CCR7−) T cells from 45.6 ± 1.3% to 66.3 ± 4.4% (Figure 1f,g). In contrast, Native and Lysate‐derived CD8+ effector and effector memory T cell populations remained equivalent upon encounter with the target U87 cells, suggesting that there was no T cell engagement. Further, to determine whether these CD8+ cells exhibited specific antitumor functionality by activating and/or degranulating upon engagement with the U87 cells, the T cells were analyzed for intracellular expression of IFNγ and TNFα, and cell surface expression of CD107a. In the T cells generated via PBNP‐PTT, both CD8+ effector T cells (39.2 ± 2.1%) and effector memory T cells (14.8 ± 6.6%) produced IFNγ and TNFα in response to target U87 cells, suggesting tumor‐specific activation (Figure 1h). In contrast, ≤1% of U87 lysate‐derived T cells generated IFNγ and TNFα in response to U87 cells, suggesting that these T cells did not activate in response to the target cells. Additionally, 46.8 ± 7.0% of CD8+ effector T cells and 21.5 ± 6.9% of CD8+ effector memory T cells expressed CD107a, suggesting degranulation in response to U87 cells, compared to 6.9 ± 1.7% and 3.8 ± 0.6% Lysate‐derived CD8+ effector and effector memory T cells, respectively (Figure 1i). These data suggest that although PBNP‐PTT and lysis of U87 cells generated equivalent populations of CD4+ and CD8+ memory T cell populations, only PBNP‐PTT‐derived CD8+ T cells activate and degranulate in response to engagement with target U87 cells, suggesting an enhanced cytotoxic capacity of this PBNP‐PTT‐derived T cell product.

### 2.2 PBNP‐PTT‐derived T cells effectively reduce orthotopic GBM tumor growth and improve survival in vivo

**FIGURE 2:**
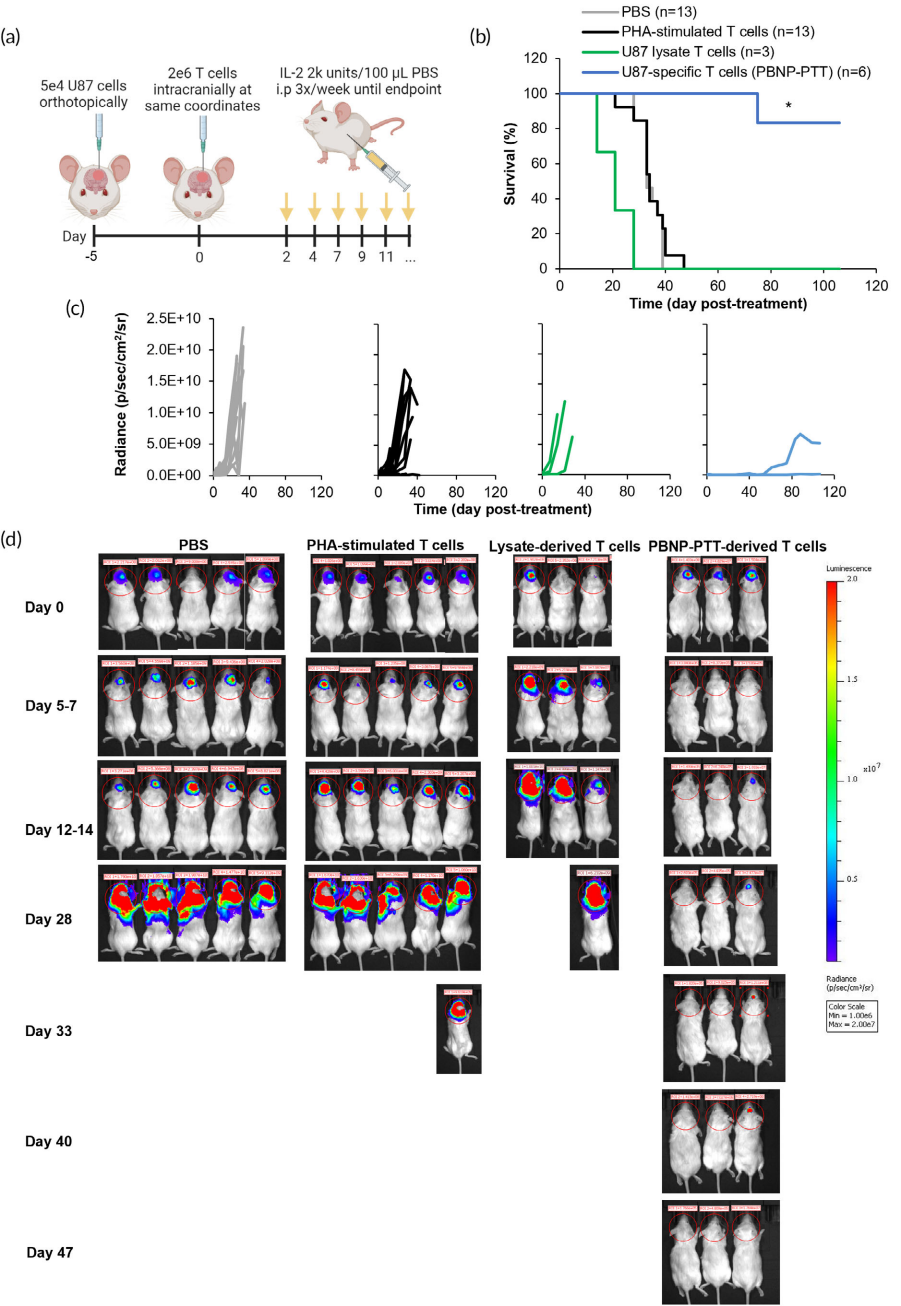
PBNP‐PTT‐derived T cells effectively reduce orthotopic GBM tumor growth and improve survival in vivo. (a) U87 cells (5e4) were orthotopically inoculated into NSG mice. Five days later, mice were intra‐cranially treated with PBS, or 2e6 PHA‐stimulated T cells, U87 lysate‐derived T cells, or U87‐specific T cells developed via PBNP‐PTT (*n* = 3–13/group; 1–3 compiled studies; 2 donors). (b) Mouse survival was tracked temporally, and tumor growth was (c) quantified and (d) visualized based on bioluminescence (a subset of animals is shown). *p < 0.05.

To investigate whether the tumor‐specific responses observed in vitro manifest in tumor control in vivo, we orthotopically inoculated NSG mice with luciferase‐transduced U87 GBM cells (Figure 2a). Upon identification of the tumor cells by bioluminescent imaging signals in the brains of U87 tumor‐bearing mice on Day 5, the mice were randomized and treated with PBS, non‐specific (PHA‐stimulated) T cells, T cells developed using U87 lysates, or U87‐specific T cells via PBNP‐PTT, by intracranial administration. U87 tumor‐bearing mice treated with PBNP‐PTT‐derived U87‐specific T cells survived significantly longer than all other groups, with 83.3% long‐term survivors (>100 days) (Figure 2b), and decreased tumor growth over time as quantified (Figure 2c) and visualized (Figure 2d) by bioluminescent imaging. Mice treated with PBS, PHA‐stimulated T cells, or Lysate‐derived T cells rapidly grew U87 tumors as indicated by increased radiance over time via bioluminescent imaging (Figure 2c,d). Mice treated with PBS, PHA‐stimulated T cells, or Lysate‐derived T cells had a median survival of 21, 34, and 33 days, respectively, significantly shorter than mice treated with PBNP‐PTT‐derived T cells (median survival was not attained in this group). These promising data suggest the advantage of PBNP‐PTT‐derived U87‐specific T cells over T cells expanded using other methods, and illustrate the first proof‐of‐concept efficacy in vivo for this novel T cell development platform.

### 2.3 PBNP‐PTT‐mediated T cell expansion generates T cells specific to several cancer cell lines

**FIGURE 3:**
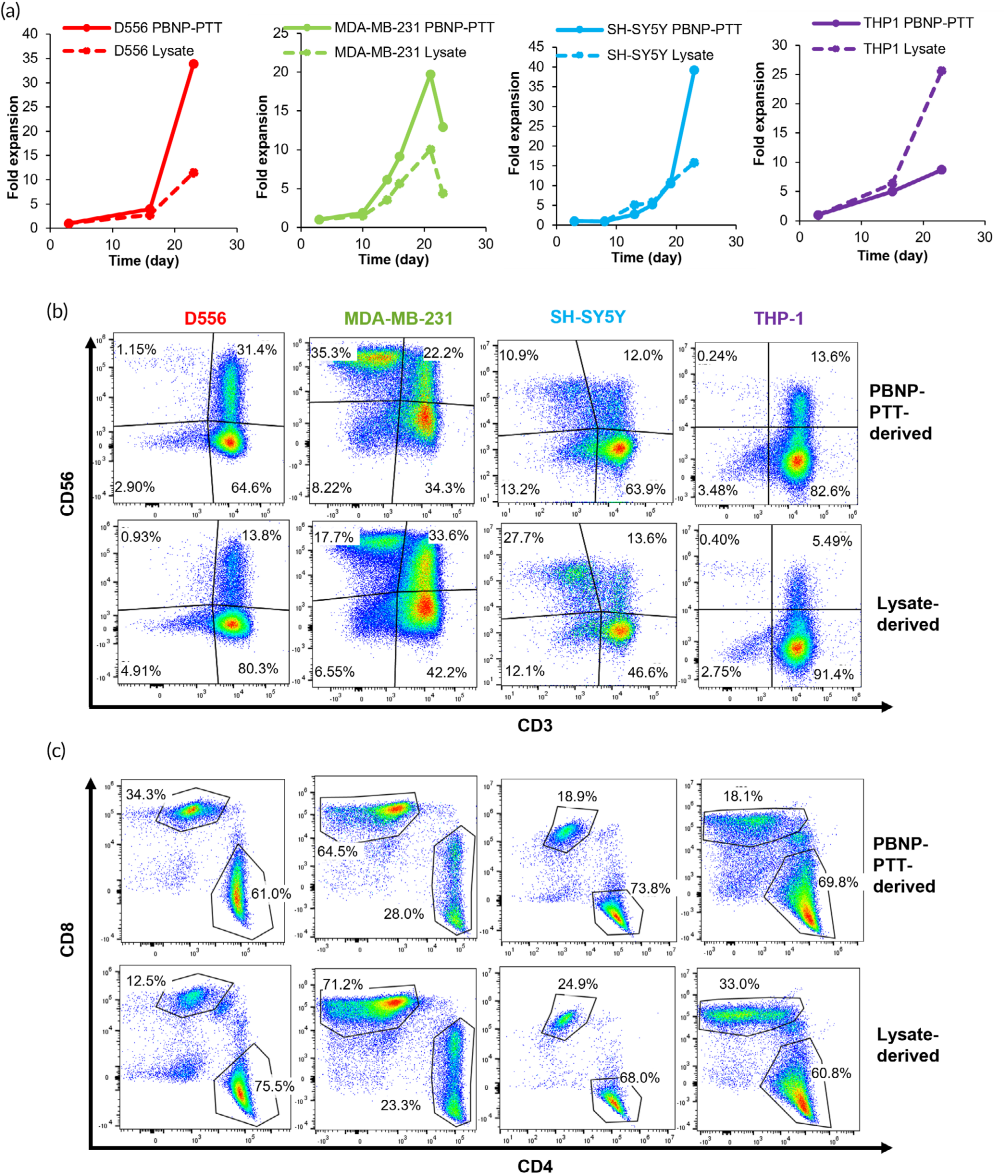
PBNP‐PTT‐mediated T cell expansion generates CD4+ and CD8+ T cells in response to several cancer cell lines. (a) T cells developed by co‐culturing with DCs primed with PBNP‐PTT‐treated tumor cells (solid lines) or lysed tumor cells (dashed lines), and their expansion, as measured by acridine orange/propidium iodide (AO/PI) staining and cell counting. (b) CD3 and CD56 expression on cell populations measured post PBNP‐PTT‐mediated or Lysate‐mediated ex vivo expansion by flow cytometry. (c) CD4 and CD8 expression on T cell populations (CD3+CD56−) measured post PBNP‐PTT‐mediated or Lysate‐mediated ex vivo expansion by flow cytometry.

To evaluate the whether the PBNP‐PTT‐mediated T cell generation strategy is robust across different cancer types, we implemented the same expansion platform to generate T cells targeting other tumor cell lines, that is, D556, a medulloblastoma cell line; MDA‐MB‐231, a breast cancer cell line; SH‐SY5Y, a neuroblastoma cell line; and THP‐1, an acute monocytic leukemia cell line. Accordingly, PBNP‐PTT or freeze–thaw lysis was individually administered to the tumor cells, which were then co‐cultured with DCs derived from healthy donors (PBMCs were matched at HLA‐A*02 to the target D556, MDA‐MB‐231, and THP‐1 cells and HLA‐A*24 to the target SH‐SY5Y cells). Then, primed DCs were added to CD14‐ cells from the corresponding donors to enable the development of tumor‐specific T cells, as detailed in the Methods section. T cells developed using the PBNP‐PTT‐based approach to target D556, MDA‐MD‐231, SH‐SY5Y, and THP‐1 cells expanded 33.9, 12.9, 39.2, and 8.7‐fold, respectively (Figure 3a). In contrast, T cells expanded using cell lysates from D556, MDA‐MD‐231, SH‐SY5Y, and THP‐1 cells expanded 11.5, 4.4, 15.8, and 25.6‐fold, respectively (Figure 3a). The expanded T cell products generated using PBNP‐PTT or lysates were analyzed for T cell phenotype by flow cytometry. PBNP‐PTT‐mediated ex vivo expansion resulted in 64.6, 34.3, 63.9, and 82.6% CD3+CD56− cells targeted to D556, MDA‐MB‐231, SH‐SY5Y, and THP‐1, respectively, which we identify as T cells (Figure 3b). Lysate‐derived cells targeting D556, MDA‐MB‐231, SH‐SY5Y, and THP‐1, comprised 80.3, 42.2, 46.6, and 91.4% T cells (Figure 3b). Of the identified T cells (CD3+CD56−), PBNP‐PTT‐derived cells comprised 61.0, 28.0, 73.8, and 69.8 CD4+ T cells and 34.3, 64.5, 18.9, and 18.1% CD8+ T cells for D556, MDA‐MB‐231, SH‐SY5Y, and THP‐1, respectively (Figure 3c). Similarly, Lysate‐derived cells targeted to D556, MDA‐MB‐231, SH‐SY5Y, and THP‐1 comprised 75.5, 23.3, 68.0, and 60.8 CD4+ T cells, and 12.5, 71.2, 24.9, and 33.0% CD8+ cells, respectively (Figure 3c), suggesting that similar populations were expanded using the two methods. Interestingly, expansion of MDA‐MB‐231‐specific cells via PBNP‐PTT or cell lysis generated notable proportions of CD3−CD56+ cells (35.3% and 17.7%, respectively), which we identify as NK cells. The presence of NK cells in the final cell product may influence downstream cytotoxicity against the target cells in a non‐specific manner.

**FIGURE 4:**
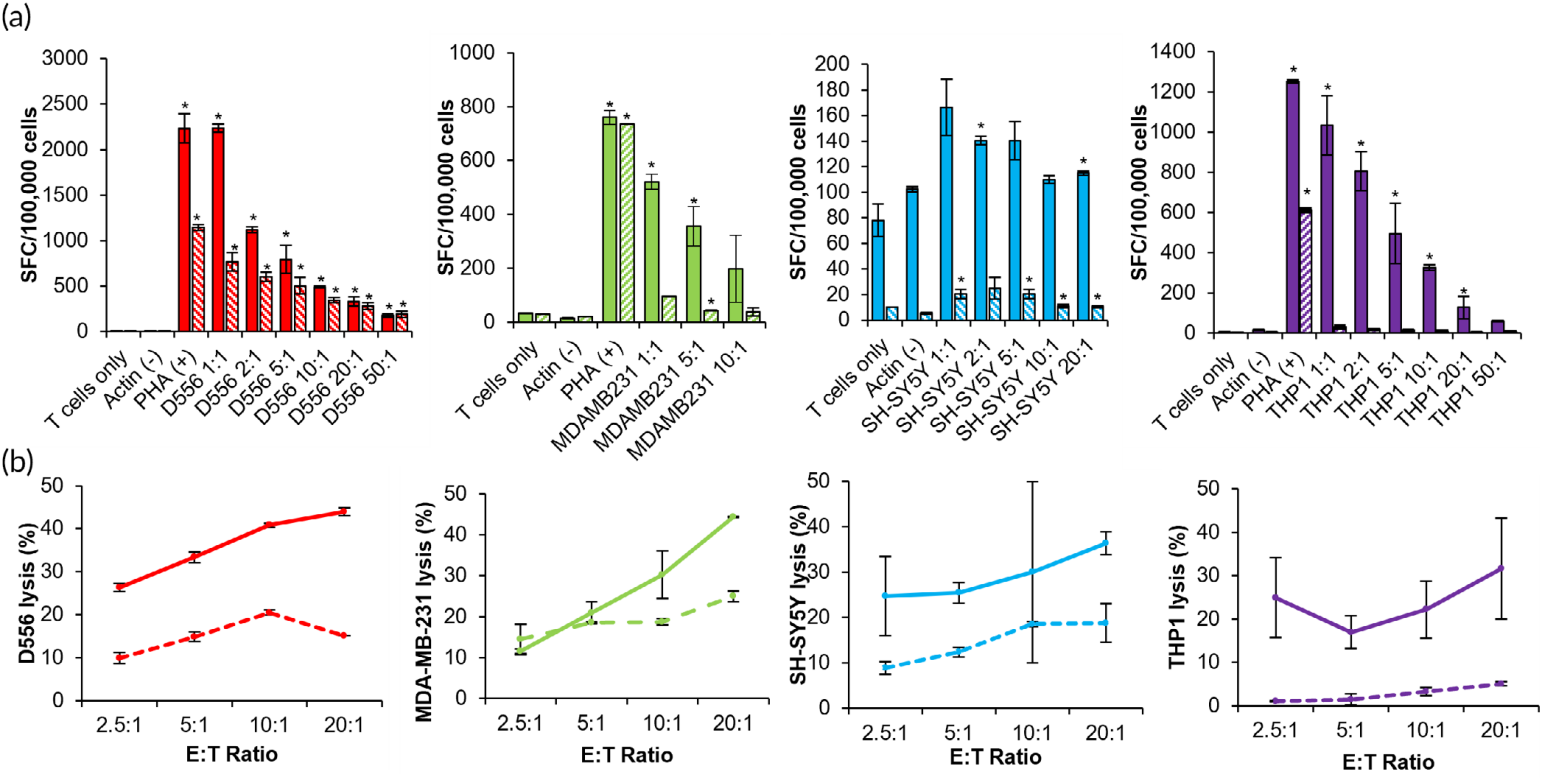
PBNP‐PTT‐mediated T cell expansion generates T cells specific to several cancer cell lines. (a) T cells developed to target several cancer cell lines via PBNP‐PTT‐mediated or Lysate‐mediated expansion were co‐cultured with tumor cells at the listed E:T ratios, and IFN‐γ release was quantified by ELISpot as spot‐forming cells (SFC) per 100,000 T cells. Media (T cells only) and Actin (1 μg/mL) were used as negative controls. PHA (1 μg/mL) was used as a positive control (removed from SH‐SY5Y plot for clarity). Values represent mean ± standard deviation (*n* = 2/group). *p < 0.05 compared to Actin. (b) T cells developed to target tumor cells via PBNP‐PTT‐mediated (solid lines) or Lysate‐mediated (dashed lines) expansion were co‐cultured with target cells at the listed E:T ratios for 4 h after 23 days (D556, MDA‐MB‐231, SH‐SY5Y) or 15 days (THP‐1). Cytotoxicity was measured by calcein release. Values represent mean ± standard deviation (*n* = 2/group).

Having determined the expansion and phenotype of PBNP‐PTT‐derived and Lysate‐derived T cells for multiple cancer types, the specificity and functionality of the resulting T cells were analyzed by co‐culture of T cells with the target tumor cells. T cells developed against all tumor types significantly (*p* < 0.05 compared to actin) secreted IFNγ in response to exposure to target cells in an E:T ratio‐dependent manner (Figure 4a). At an E:T ratio of 1:1, 167 to 2238 T cells secreted IFNγ in response to target cells, depending on the tumor type. At E:T ratios of 2:1, 5:1, 10:1, 20:1, or 50:1, T cells secreted IFNγ in a dose‐dependent manner. In contrast, at all E:T ratios tested, fewer Lysate‐derived T cells for the same tumor cell lines secreted IFNγ in response to target cells, suggesting that these T cell products are less specific and responsive to the target cells. We next evaluated whether IFNγ secretion from PBNP‐PTT‐derived tumor‐specific T cells corresponded with an increased cytotoxicity toward target cells. Consequently, T cells manufactured using PBNP‐PTT were harvested on day 23 (i.e., D556, MDA‐MB‐231, SH‐SY5Y or Day 15 (THP‐1) and co‐cultured with target tumor cells. At E:T ratios of 2.5:1, 5:1, 10:1, and 20:1, T cells expanded using PBNP‐PTT killed 11.6%–26.3%, 17.0%–33.3%, 22.2%–40.8%, and 31.7%–44.3% of target tumor cells in a co‐culture after 4 hours, respectively, depending on the tumor type (Figure 4b). At the same E:T ratios, T cells expanded using tumor cell lysis killed 1.1%–14.5%, 1.5%–18.6%, 3.3%–20.4%, and 5.1%–24.9%, depending on the tumor type, markedly lower than those developed using PBNP‐PTT. These data suggest that the PBNP‐PTT‐mediated T cell expansion strategy is eliciting a tumor‐specific T cell response and may be broadly applicable to different tumor types.

## 3 DISCUSSION

The studies presented in this paper illustrate the potential applicability of a PBNP‐PTT‐mediated T cell expansion platform ex vivo to develop personalized ATCT products for treating patients with cancer. First, we described the phenotype of T cells expanded via PBNP‐PTT as compared to Lysate‐derived T cells and native CD14‐ cells from the same donors (Figure 1). PBNP‐PTT‐derived and Lysate‐derived T cells both comprised a mixed population of CD4+ and CD8+ T cells, which expressed statistically identical markers of antigen experience and memory. Notably, PBNP‐PTT expanded a significantly higher percentage of CD3+CD56+ cells, which may represent activated T cells or an NKT cell‐like population.^56^, ^57^ Identification of the invariant TCR α‐chain is necessary before characterizing this population as NKT cells.^58^, ^59^

We focused our functional T cell studies on the CD8+ T cell subset, as the healthy donor PBMCs used in the manufacture were matched to the target cells at HLA‐A*02. As such, the T cells specific to the target tumor cells should be HLA‐A*02 restricted within the CD8+ population. Importantly, despite Lysate‐derived and PBNP‐PTT‐derived T cells expressing similar phenotypic characteristics, we found that their function in response to the target U87 cells differed dramatically. The CD8+ effector and effector memory T cell populations within PBNP‐PTT‐derived T cells expanded significantly upon interaction with the target cells, suggesting tumor cell specificity and T cell activation. In contrast, the CD8+ effector and effector memory subsets within Lysate‐derived T cells remained statistically identical before and after co‐culture with the target cells. Furthermore, these PBNP‐PTT‐derived CD8+ effector and effector memory T cells secreted IFNγ and TNFα, and expressed CD107a on their surface, upon co‐culture with the target tumor cells, suggesting T cell engagement, activation, and downstream effector function,^60^, ^61^, ^62^ in contrast to Lysate‐derived T cells and CD14− cells which did not exhibit these markers. These data highlight the benefit of using PBNP‐PTT, over other cell death strategies (e.g., cell lysis), as a means to generate multiple antigens for tumor‐specific T cell expansion. We have previously demonstrated that PBNP‐PTT facilitates ICD in cell and animal models of cancer,^12^, ^18^, ^20^ and thus may facilitate enhanced engagement with DCs for increased antigen uptake and presentation, which may be driving the outcomes observed herein.

To move toward clinical translation of a novel PBNP‐PTT‐derived ATCT, we next evaluated the antitumor functionality of the T cells in an orthotopic GBM model in vivo. PBNP‐PTT‐derived T cells effectively treated established GBM tumors and significantly improved animal survival, as compared to Lysate‐derived T cells, PHA‐stimulated T cells, or PBS controls (Figure 2). Because these T cells and tumor cells were sourced from humans, the in vivo studies were performed in immunocompromised NSG mice, and thus, in the absence of an endogenous immune system. Therefore, these studies do not address in the impact of host immune cells on the adopted T cells (e.g., rejection of the therapeutic T cells, host‐versus‐graft disease), which may be present in a fully immunocompetent model. Nor do these studies evaluate the impact of the adopted T cells on the host immune system, which may lead to endogenous T cell engagement with the tumor through epitope spreading,^63^, ^64^ or toxicity (e.g., graft‐versus‐host disease).

Additionally, we have presented proof‐of‐concept in vivo in one disease model (GBM) using an intracranial injection of the T cells. The intracranial route of administration is warranted for GBM and other brain malignancies (e.g., medulloblastoma) because of their anatomical location.^41^, ^65^, ^66^ However, different routes of administration will be required for other tumor types, such as breast cancer, neuroblastoma, and acute monocytic leukemia investigated here. For the solid tumors (breast, neuroblastoma), we envision either intravenous or intratumoral administration of the ATCT^67^, ^68^; for acute monocytic leukemia and other hematological malignancies, we envision intravenous administration of the ATCT.^69^, ^70^

Finally, this work suggests that PBNP‐PTT represents a tumor‐agnostic T cell expansion platform that is applicable across tumor types (Figures 3 and 4). PBNP‐PTT‐derived T cells were generated to target medulloblastoma, breast cancer, neuroblastoma, and acute monocytic leukemia cell lines. Interestingly, the breast cancer (MDA‐MB‐231)‐specific PBNP‐PTT‐derived T cells expanded the least (4.4‐fold) over the 23‐day expansion period (Figure 3a), and exhibited the highest proportion of NK cells (35.3%, as identified as CD3−CD56+). NK cells can also secrete IFNγ, so it is possible that both T cells and/or NK cells were responsible for the dose‐dependent IFNγ observed in response to the target cells (Figure 4a). We will consider sorting the final product for CD3+ cells to eliminate this NK cell fraction to ensure a purer T cell population. However, although NK cells are primarily involved in innate immunity, they may play a role in adaptive immunity as well^71^; further studies are warranted to describe the role of NK cells expanded using the PBNP‐PTT platform.

Overall, these studies lay the foundation for further development of the PBNP‐PTT‐based T cell development platform. Ongoing studies will test the PBNP‐PTT platform in xenograft models of other cancer types beyond GBM, and the findings will be validated using PBMCs from several additional healthy donors.

## 4 MATERIALS AND METHODS

### 4.1 Cell lines and cell culture

U87, luciferase‐transduced U87, MDA‐MB‐231, SH‐SY5Y, and THP‐1 cells were obtained from the American Type Culture Collection (ATCC; Manassas, VA). D556 was provided by Dr. Cruz at Children's National Hospital. U87 and D556 cells were grown in Minimum Essential Medium Eagle (MEM media; Millipore Sigma, Burlington, MA) supplemented with 10% heat‐inactivated fetal bovine serum (FBS; Cytiva, Marlborough, MA), 1% Glutamax (Gibco, Waltham, MA) and 1% penicillin–streptomycin (Gibco). MDA‐MB‐231 cells were grown in Dulbecco's Modified Medium (DMEM; Gibco) supplemented with 10% heat‐inactivated FBS (Cytiva) and 1% penicillin–streptomycin (Gibco). SH‐SY5Y cells were grown in a 1:1 mixture of MEM media and F12‐K media (Gibco), supplemented with 10% heat‐inactivated FBS (Cytiva) and 1% penicillin–streptomycin (Gibco). THP‐1 cells were grown in RPMI‐1640 media (Gibco) supplemented with 0.05 mM 2‐mercaptoethanol (Sigma) and 10% FBS (Cytiva).

Cells were expanded as per the specifications provided by the supplier. Healthy donor peripheral blood leukopaks were purchased from AllCells (Alameda, CA) with the corresponding HLA reports. Peripheral blood mononuclear cells (PBMCs) were matched at the HLA‐A*02 antigen expressed by D556, MDA‐MB‐231, and THP‐1 cells and HLA‐A*24 antigen expressed by the SH‐SY5Y cells. As blood was sourced from a commercial vendor, healthy donors had been de‐identified prior to our receipt to ensure privacy, and are thus not subject to IRB compliance. PBMCs were isolated by density gradient centrifugation using lymphocyte‐separation media (LSM; Corning, Corning, NY). PBMCs were cryopreserved for future use. Dendritic cells (DCs) were cultured in DC media (CellGenix, Freiburg im Breisgau, Germany) supplemented with 1% Glutamax (Gibco). T cells were cultured in 46.5% Click's media (Millipore Sigma), 46.5% RPMI‐1640 (Gibco), 5% human AB serum (Gemini Bio‐products, Sacramento, CA), 1% Glutamax (Gibco) and 1% penicillin streptomycin (Gibco).

### 4.2 PBNP‐PTT treatment of tumor cell lines

PBNPs used for PTT were synthesized as described previously.^72^, ^73^ Potassium hexacyanoferrate (II) trihydrate (K_4_[Fe(CN)_6_]·3H_2_O) and iron (III) chloride hexahydrate (Fe(Cl)_3_·6H_2_O) were purchased from Millipore Sigma. Briefly, an aqueous solution of 1.0 mM FeCl_3_·6H_2_O in 20 mL of deionized (DI) water was added under vigorous stirring to an aqueous solution containing 1.0 mM of K_4_Fe(CN)_6_·3H_2_O in 20 mL of DI water. After stirring for 15 min, the precipitate was isolated by centrifugation in equal parts DI water and acetone (10,000 × *g* for 10 min) at room temperature, and rinsed by sonication (5 s, 40% amplitude) in DI water using a Q500 sonicator (QSonica LLC, Newton, CT). The isolation and rinsing steps were repeated three times before the nanoparticles were resuspended by sonication in DI water. Quality control of the nanoparticles was performed as previously described using dynamic light scattering, UV–Vis–NIR spectrophotometry, and zetametry.^12^, ^21^ To administer PBNP‐PTT to the tumor cells, five million tumor cells were suspended in 500 μL of DC media with 0.15 mg/mL PBNPs. The samples were then illuminated using a NIR laser (808 nm; Laserglow Technologies, Toronto, Canada) for 10 min at 1.5 W. The laser power used was confirmed using a power meter (Thorlabs, Newton, NJ). PBNP‐PTT‐treated cells were then mixed using a pipette and added directly to DCs in DC media in a 1:1 ratio of DC: tumor cells (1e6 DCs and 1e6 tumor cells) in a 24‐well plate, immediately after PBNP‐PTT was completed. The DC and tumor cell co‐culture was then incubated at 37°C overnight.

### 4.3 T cell expansion protocol

T cell lines were generated from PBMCs matched at HLA‐A*02 for U87, D556, MDA‐MB‐231, or THP‐1 cells or at HLA‐A*24 for SH‐SY5Y cells using monocyte‐derived dendritic cells (DCs) based on a previously established protocol.^21^, ^74^ Two unique donors were used to generate U87‐specific T cells. Briefly, monocytes were isolated using a CD14 isolation MACS MicroBeads kit (Miltenyi Biotec, Bergisch Gladbach, North Rhine‐Westphalia, Germany) per the manufacturer's instructions. The isolated CD14 positive (CD14+) cells were cultured in DC media in the presence of IL‐4 (1000 U/mL) and granulocyte‐macrophage colony‐stimulating factor (GM‐CSF; 800 U/mL) (R&D Systems, Minneapolis, MN). The CD14 negative (CD14−) fraction was cryopreserved for later use. Two days later (day 2), the CD14+ cells (now termed DCs) from each donor were pulsed with tumor cells subjected to PBNP‐PTT as described above and previously^20^, ^21^ at 1.5 W laser power, tumor cell lysates generated using multiple freeze–thaw cycles using a dry ice‐ethanol mixture and a 37°C water bath.^18^, ^75^ The PBNP‐PTT‐treated tumor cells or tumor cell lysates were added to DCs at a ratio of 1:1. On the same day that the treated tumor cells were added to DCs (day 2), DCs were matured with GM‐CSF (800 U/mL), TNF‐α (10 ng/mL), IL‐1β (10 ng/mL), IL‐4 (1000 U/mL), IL‐6 (100 ng/mL), IFNγ (100 U/mL) (R&D Systems), and lipopolysaccharide (LPS; 30 ng/mL; Millipore Sigma) overnight. The following day (day 3), the cryopreserved CD14− PBMCs were thawed and stimulated with the primed and harvested DCs at a 1:5 (DC:T cell) ratio in T cell media supplemented with IL‐6 (100 ng/mL), IL‐7 (10 ng/mL), IL‐12 (10 ng/mL), and IL‐15 (5 ng/mL) (R&D Systems). These cells were incubated at 37°C and were expanded and fed with IL‐6 (100 ng/mL), IL‐7 (10 ng/mL), and IL‐15 (5 ng/mL) (R&D Systems) as needed. On day 13, frozen PBMCs from the same donor were thawed, and CD14 isolation was performed as described above. On day 15, DCs were again pulsed with tumor cells subjected to PBNP‐PTT or cell lysis at a ratio of 1:1 (tumor cell:DC), and matured as described above. The following day (day 16) these matured DCs were added to the ongoing T cell culture at a ratio of 1:5 (DC:T cell) in the presence of IL‐7 (10 ng/mL) (R&D Systems) and IL‐2 (50 U/mL) (Proleukin; Clinigen Group, UK). T cells were fed and split with fresh media and IL‐15 (5 ng/mL) (R&D Systems) when necessary. On day 23 (day 15 for THP‐1), cells were harvested for phenotyping and functional assays. Control PHA‐stimulated T cells were generated using CD14− cells from the same donor PBMCs. CD14− cells were stimulated with lectin, Phaseolus vulgaris (PHA; 5 mg/mL; Millipore Sigma) on day 1, and cultured for 11 days in RPMI‐1640 (Gibco) supplemented with 5%–10% human AB serum (Gemini Bioproducts), 1% Glutamax (Gibco) and 1% penicillin streptomycin (Gibco) and IL‐2 (100 U/mL; Clinigen Group). Expansion was quantified by harvesting and counting with acridine orange/propidium iodide (AO/PI) cell viability kit (Logos Biosystems, Korea) on a LUNA‐FL automated cell counter (Logos Biosystems) periodically throughout the development scheme. T cell phenotype, specificity, and cytotoxic functionality was measured by flow cytometry, ELISpot, and calcein AM cytotoxicity assays, respectively, as described below.

### 4.4 T cell phenotyping

T cells were harvested on day 23 of the expansion protocol and assessed for viability and phenotypic markers. The cells were stained with Zombie green fixable viability dye (Biolegend, San Diego, CA), blocked with human TruStain Fc block (Biolegend), and stained with fluorescent antibodies from Biolegend against human CD3 (clone HIT3a), CD56 (clone HCD56), CD4 (clone RPA‐T4), CD8 (clone SK1), CD45RA (clone HI100), CD45RO (clone UCHL1), CD62L (clone DREG‐56), CCR7 (clone G043H7), or CD107a (clone H4A3). For intracellular identification of cytokines, cells were fixed and permeabilized using the BD Cytofix/Cytoperm Plus Fixation/Permeabilization Solution Kit with BD GolgiStop (BD Biosciences, Franklin Lakes, NJ) and stained with fluorescent antibodies against IFNγ (clone B27) or TNFα (clone MAb11). Flow cytometry was performed on either the Beckman Coulter Cytoflex (Brea, CA) or BD FACSCelesta (Franklin Lakes, NJ) flow cytometer and cytometric analysis was performed using FlowJo software.

### 4.5 T cell specificity analysis

T cell specificity was determined by IFNγ ELISpot. Multiscreen HTS IP filter plates (Millipore Sigma) were coated with an IFNγ capture antibody (clone 1‐D1K; Mabtech, Stockholm, Sweden). On the following day, the target cells were collected from 37°C incubation. T cells were either plated alone or in presence of target cells at varied effector to target cell (E:T) ratios (i.e., 1:1, 2:1, 5:1, 10:1, 20:1, 50:1), keeping the number of T cells constant at 100,000 cells per well and varying the number of target cells. Actin (1 μg/mL) was administered to the T cells as a negative control (JPT peptide technologies, Berlin, Germany); 1 μg/mL PHA (Millipore Sigma) was administered to the T cells as a positive control for T cell activation. Cells were incubated at 37°C and the ELISpot plate was developed 24 h later using the manufacturer's protocol. The number of IFNγ spot‐forming units were quantified by Zellnet Consulting (Fort Lee, NJ). The frequency of tumor‐specific T cells was expressed as spot‐forming cells (SFC) per 100,000 cells.

### 4.6 T cell cytotoxicity analysis

To assess the cytotoxicity of T cells against target cells, we conducted a calcein release assay. Target cells were labeled with calcein AM (ThermoFisher Scientific, Waltham, MA) for 30 min at 37°C. Spontaneous and total release of calcein dye from the target cells was measured using a SpectraMax i3X microplate reader (Molecular Devices, San Jose, CA) by treating the cells with media or 2% Triton X‐100 (Millipore Sigma), respectively.^76^, ^77^ Expanded T cells were resuspended at different concentrations and were mixed with the target cells to generate E:T ratios of 2.5:1, 5:1, 10:1, or 20:1. Target cell concentration remained constant in all E:T ratios (10,000 target cells per well), and T cells were varied (10,000–500,000 T cells per well depending on the E:T ratio). To quantify T cell cytotoxicity‐based calcein release, the co‐cultures of T cells and tumor cells were incubated for 4 h at 37°C whereupon the supernatants were harvested and analyzed for calcein dye release using the SpectraMax i3X microplate reader. Specific lysis was calculated using the formula: (sample measurement − spontaneous release)/(total release − spontaneous release)*100.

### 4.7 Animals

NOD.Cg‐Prkdc scid Il2rg tm1Wjl/SzJ (NSG) mice (female, 5–8 weeks old) were purchased from Jackson Laboratory (Bar Harbor, ME) and the animal protocols approved by the Institutional Animal Care and Use Committee at Children's National Hospital, Washington, DC. Luciferase‐transduced U87 cells (5e4) were orthotopically inoculated into the brains of mice using rodent stereotactic surgery. On day 5, the mice exhibited measurable bioluminescence signal in the brains and were intracranially treated with PBS, 2e6 PHA‐stimulated T cells, 2e6 U87 lysate‐derived T cells, or 2e6 U87‐specific T cells developed via PBNP‐PTT (*n* = 3–5/group; 1–3 compiled studies) at the same location. Mouse survival was tracked temporally, and tumor growth was quantified and visualized based on bioluminescence on an IVIS Lumina 100 (PerkinElmer, Waltham, MA), and images were scaled to the same minimum and maximum photon distribution prior to analysis. Animals were injected with 150 mg/kg D‐Luciferin (GoldBio, St. Louis, MO) 5 min prior to imaging with the IVIS.

### 4.8 Statistics

Statistically significant differences between groups were determined using pairwise *t*‐tests or the log‐rank test in Microsoft Excel (Redmond, WA). Values were considered statistically significantly different when *p* values were less than 0.05.

## 5 CONCLUSIONS

The data presented herein describe a novel and multi‐targeted tumor‐specific T cell development scheme using Prussian blue nanoparticle‐based photothermal therapy. This ATCT manufacturing platform was applied across tumor types using cell lines in both hematological and solid tumors for the adult and pediatric populations. The ATCT phenotyping analysis uncovered the critical role of CD8+ T cells, particularly CD8+ effector and CD8+ effector memory T cells, in driving the observed antitumor responses in a cellular model of GBM. The pilot animal study suggested the in vivo efficacy of the tumor‐specific T cells in the setting of GBM. Finally, preliminary studies revealed the potential of the PBNP‐PTT‐based ATCT development platform to expand T cells specific to tumor cell lines beyond GBM, that is, medulloblastoma, breast cancer, neuroblastoma, and acute monocytic leukemia. Further studies are ongoing to validate these findings in additional donors, as well as in the autologous setting using patient‐derived tumor and blood samples.

## AUTHOR CONTRIBUTIONS

**Elizabeth E. Sweeney**: Conceptualization, methodology, formal analysis, data curation, writing—original draft, writing—review & editing, visualization, supervision, project administration, funding acquisition; **Palak Sekhri**: Methodology, validation, investigation, formal analysis; **Nethaji Muniraj**: Methodology, validation, investigation; **Jie Chen**: Methodology, validation, investigation, formal analysis; **Sally Feng**: Methodology, validation, investigation; **Joshua Terao**: Methodology, validation, investigation; **Samantha J. Chin**: Methodology, validation, investigation; **Danielle E. Schmidt**: Validation, investigation; **Catherine M. Bollard**: Conceptualization, supervision; **Conrad Russell Y. Cruz**: Conceptualization, methodology, formal analysis, data curation, supervision; **Rohan Fernandes**: Conceptualization, methodology, resources, data curation, writing—original draft, writing—review & editing, supervision, project administration, funding acquisition.

## CONFLICT OF INTEREST STATEMENT

Catherine M. Bollard (CMB) is a past scientific advisory board member for NexImmune and Repertoire Immune Medicines, both antigen‐specific T cell companies. CMB has stock or ownership in Cabaletta Bio, Catamaran Bio, and NexImmune. Elizabeth E. Sweeney (EES) and Rohan Fernandes (RF) are co‐founders of ImmunoBlue, a biotechnology company focused on developing PBNP‐based nanoimmunotherapies. EES, Palak Sekhri, C. Russell Y. Cruz, and RF have jointly filed a patent application protecting the work described in this manuscript.

### PEER REVIEW

The peer review history for this article is available at https://www.webofscience.com/api/gateway/wos/peer-review/10.1002/btm2.10639.

## Data Availability

The data that support the findings of this study are available from the corresponding author upon reasonable request.
